# On the lineage of *Aspergillus fumigatus* isolates in common laboratory use

**DOI:** 10.1093/mmy/myaa075

**Published:** 2020-09-17

**Authors:** Margherita Bertuzzi, Norman van Rhijn, Sven Krappmann, Paul Bowyer, Michael J Bromley, Elaine M Bignell

**Affiliations:** Manchester Fungal Infection Group, Faculty of Biology, Medicine and Health, The University of Manchester, Manchester Academic Health Science Centre, Core Technology Facility, Manchester, UK; Lydia Becker Institute of Immunology and Inflammation, Biology, Medicine and Health. The University of Manchester, Manchester Academic Health Science Centre, Manchester, UK; Manchester Fungal Infection Group, Faculty of Biology, Medicine and Health, The University of Manchester, Manchester Academic Health Science Centre, Core Technology Facility, Manchester, UK; Lydia Becker Institute of Immunology and Inflammation, Biology, Medicine and Health. The University of Manchester, Manchester Academic Health Science Centre, Manchester, UK; Institute of Clinical Microbiology, Immunology and Hygiene, University Hospital Erlangen, Friedrich-Alexander University Erlangen-Nürnberg, Germany; Manchester Fungal Infection Group, Faculty of Biology, Medicine and Health, The University of Manchester, Manchester Academic Health Science Centre, Core Technology Facility, Manchester, UK; Lydia Becker Institute of Immunology and Inflammation, Biology, Medicine and Health. The University of Manchester, Manchester Academic Health Science Centre, Manchester, UK; Manchester Fungal Infection Group, Faculty of Biology, Medicine and Health, The University of Manchester, Manchester Academic Health Science Centre, Core Technology Facility, Manchester, UK; Lydia Becker Institute of Immunology and Inflammation, Biology, Medicine and Health. The University of Manchester, Manchester Academic Health Science Centre, Manchester, UK; Manchester Fungal Infection Group, Faculty of Biology, Medicine and Health, The University of Manchester, Manchester Academic Health Science Centre, Core Technology Facility, Manchester, UK; Lydia Becker Institute of Immunology and Inflammation, Biology, Medicine and Health. The University of Manchester, Manchester Academic Health Science Centre, Manchester, UK; MRC Centre for Medical Mycology, University of Exeter, Geoffrey Pope Building, Stocker Road, Exeter EX4 4QD, UK

**Keywords:** *Aspergillus fumigatus*, strain, lineage, isolate

## Abstract

The origin of isolates routinely used by the community of *Aspergillus fumigatus* researchers is periodically a matter of intense discussion at our centre, as the construction of recombinant isolates have sometimes followed convoluted routes, the documentation describing their lineages is fragmented, and the nomenclature is confusing. As an *aide memoir*, not least for our own benefit, we submit the following account and tabulated list of strains (Table 1) in an effort to collate all of the relevant information in a single, easily accessible document. To maximise the accuracy of this record we have consulted widely amongst the community of Medical Mycologists using these strains. All the strains described are currently available from one of these organisations, namely the Fungal Genetics Stock Centre (FGSC), FungiDB, Ensembl Fungi and The National Collection of Pathogenic Fungi (NCPF) at Public Health England. Display items from this manuscript are also featured on FungiDB.

**Lay abstract:**

We present a concise overview on the definition, origin and unique genetic makeup of the *Aspergillus fumigatus* isolates routinely in use by the fungal research community, to aid researchers to describe past and new strains and the experimental differences observed more accurately.

## Sequenced *A. fumigatus* isolates

**Figure 1.**
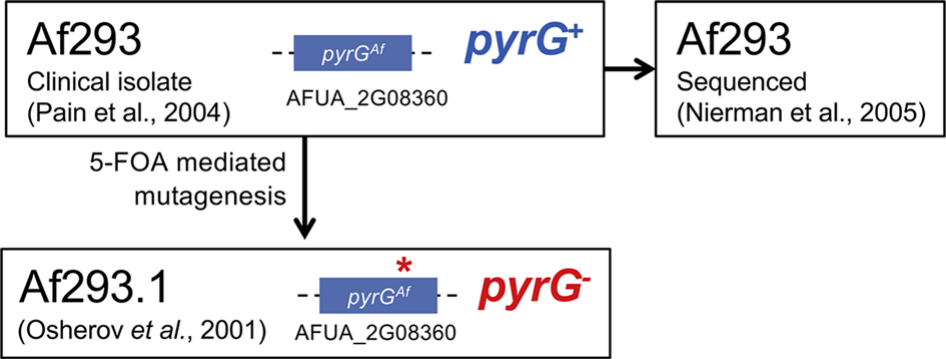
Af293 and derivative thereof. Diagram depicting provenance and scientific use of Af293 strains. The uridine/uracil requirements and the genomic organization of the *pyrG* locus is indicated for each isolate within this lineage.

**Figure 2.**
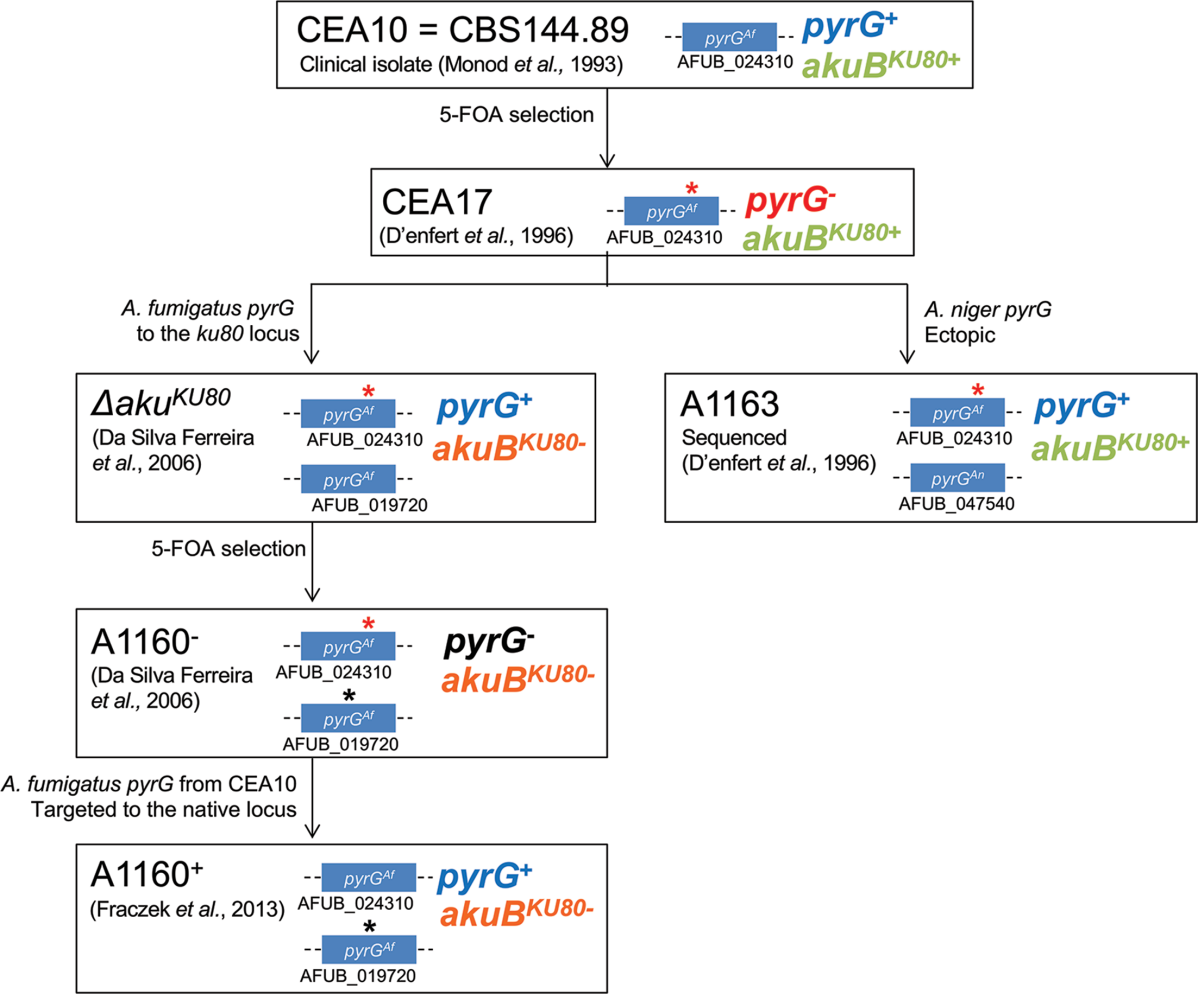
CEA10 and derivatives thereof. Diagram depicting provenance and scientific use of CEA10 strains. The uridine/uracil requirements and the genomic organization of the *pyrG* and *akuB*^KU80^ loci is indicated for each isolate within this lineage.

Fully annotated genomic sequences for two *A. fumigatus* strains Af293 and A1163 (Figs. 1 and 2) are available at FungiDB (https://fungidb.org/fungidb). FungiDB also compiles gene, protein, and chromosome sequence information about these and other *Aspergillus* species with descriptions and classifications of their biological roles, molecular functions, and subcellular localizations, while also offering tools for analyses and comparison of sequences and links to literature information.

As of the beginning of 2020, Ensembl Fungi (http://fungi.ensembl.org/index.html) and NCBI (https://www.ncbi.nlm.nih.gov/genome/genomes/18) contain the genomic sequences of 5 and 14 different *A. fumigatus* isolates, respectively, and genomic data for many hundreds more *A. fumigatus* isolates have been deposited into genomic databases including the NCBI bioprojects database (https://www.ncbi.nlm.nih.gov/bioproject/), which lists more than 100 entries. Genome sequences for a cohort of 66 isolates were curated and characterized in a recent study of genetic diversity in secondary metabolism gene clusters,^1^ and for a cohort of 24 clinical and environmental isolates in a recent study of azole resistance.^2^

## Af293 and derivatives

Af293 is a clinically derived strain, which was isolated in 1993 from a lung biopsy taken postautopsy from a neutropenic patient that received treatment for rheumatoid arthritis, developing severe sepsis syndrome. Microscopy of the tissue revealed septate hyphae consistent with aspergillosis. From the biopsy, the Public Health Laboratory at the Royal Shrewsbury Hospital grew a culture yielding *A. fumigatus*. Samples were sent to Hope Hospital where it was assigned the name Af293. In 1998, the isolate was sent to the National Collection of Pathogenic Fungi (NCPF) and assigned NCPF7367.^3^ The strain was sequenced in 2005 making it the first *A. fumigatus* isolate with a publicly available full genome sequence.^4^ At the present time, the Af293 genome remains the only genome sequence of the species to have been fully physically reconstructed as chromosomes. The same study furnished the community with an oligomer-based microarray technology, which was subsequently used for multiple transcriptomic analyses.^5–12^

Organized on eight chromosomes, the Af293 genome spans 29.4 Mb. While the sequence of Af293 is over 97% identical to the A1163 genome, significant diversity of genetic content was found in the telomeric and subtelomeric regions.^13^ Comparative analysis of a larger set of genomes revealed that Af293 belongs to a different clade than the CEA10/A1163 lineage^14^ (where A1163 is the sequenced derivative isolate of the clinical isolate CEA10, see Fig. 2). When compared to the CEA10/A1163 lineage Af293 was found to exhibit a slower growth rate on solid *Aspergillus* minimal media, while having a faster growth rate in liquid media and heightened sensitivity to hypoxic conditions as well as being less pathogenic than CEA10 in a triamcinolone murine model of infection.^15^ Similar attenuation of virulence was reported from a zebrafish model of infection, seemingly due to differences in neutrophil and macrophage mediated killing of *A. fumigatus*.^16^ Distinct mechanistic bases for leukocyte recruitment in response to Af293 or CEA10 infection could also be correlated with strain-specific immune responses *in vivo*.^17^

To facilitate a genetic screen for itraconazole resistance, a derivative strain Af293.1 was generated by Osherov et al.^18^ by exposing Af293 to 4-nitroquinoline 1-oxide, followed by selection on uracil and uridine supplemented media containing 5-fluoro-orotic acid (5-FOA) to select for loss of function mutations in the *pyrG* (*AFUA_2G08360*) gene (Fig. 1).

## CEA10 and derivatives

CEA10 is a clinically derived strain isolated in the early ’90s from a patient with invasive aspergillosis.^19,20^ Due to the use of CEA10 as a progenitor strain in which non-homologous end joining mutants have been constructed^21^ strains in the CEA10 lineage (Fig. 2) have been extensively utilized by many research groups. CEA10 is more pathogenic in murine models of infection than the Af293 and ATCC46645 isolates.^5,15^

In 1996 d'Enfert et al. utilized CEA10 to develop a *pyrG*-blaster tool with which to elicit iterative gene deletions in a single strain.^22^ The *pyrG*-blaster cassette consisted of the *Aspergillus niger pyrG* gene flanked by a direct repeat that encodes the neomycin phosphotransferase of transposon Tn5. In order to produce a pyrimidine auxotroph with which to select for insertions of the *pyrG*-blaster, 4-nitroquinoline-N-oxide mutagenesis and 5-FOA selection were implemented yielding a pyrimidine auxotrophic derivative of CEA10, designated CEA17.

**Figure 3.**
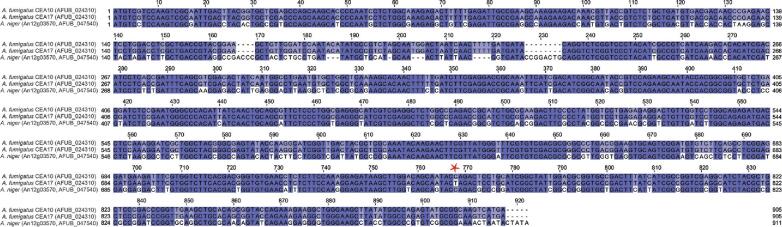
Alignment of the DNA sequences of *A. fumigatus pyrG* gene from CEA10 and CEA17 and *A. niger pyrG* as present in A1163. The asterisk indicates the C nucleotide in position 1413 that has changed into a T in the CEA17 strain, leading to the transition from glutamine to a premature stop codon. This nucleotide change can be used as a diagnostic marker to differentiate strains within the CEA10 lineage. *A. fumigatus pyrG* and *A. niger pyrG* share 77.16% identity. As *A. niger pyrG* is ectopically integrated in the genome of *A. fumigatus* A1163, the presence of this gene can be used as a diagnostic marker to differentiate *A. fumigatus* A1163 from the rest of the isolates within the CEA10 lineage.

CEA17, which was found to harbor a single point mutation in the gene that encodes orotidine-5′-phosphate decarboxylase (*pyrG, AFUB_024310*) resulting in the generation of a premature stop codon (Fig. 3, C to T transition at + 1413 from the start codon), provided the basis for subsequent development of a first *pyrG*-dependent homologous transformation system for *A. fumigatus*.^23^

A CEA17 derivative, harboring a functional copy of the *A. niger pyrG* (*An12g03570*, named *AFUB_047540* in the annotation of the A1163 genome), and designated A1163 was subsequently sequenced at the J. Craig Venter Institute, in collaboration with Celera Genomics.^13^ Although the lineage of the A1163 isolate is irrefutably confirmed by genetic analysis, the precise origin of this laboratory isolate remains unclear.

The A1163 genome is 29.2 Mb in size. The genome is believed to be organized onto eight chromosomes but, unlike the Af293 genome, has not been fully physically reconstructed and is reported as 223 contig sequences.

*∆akuB* ^KU80^ is a CEA17 derivative lacking the *ku80* (*AFUB_019720*) gene, also named *akuB*^KU80^^24^ and was generated to facilitate molecular manipulations of *A. fumigatus*. The *akuB*^KU80^ gene encodes one component of the heterodimeric Ku protein complex, which is an essential mediator of the nonhomologous end joining (NHEJ) DNA repair pathway. The *∆akuB*^KU80^ strain exhibits dramatically heightened rates (80% compared to 4% in wild type strains) of targeted genomic integration of exogenous DNA by homologous recombination.^24^ The *akuB*^KU80^ gene was replaced with a *zeo-pyrG* cassette, that contains the *A. fumigatus pyrG* gene (from isolate ATCC46645) amplified from the pCDA21 plasmid^25^ and the *Streptoalloteichus hindustanus* Sh *ble* gene conferring resistance to the antibiotic zeocin^26^ under the control of the EM7 promoter from plasmid pEM7-zeo (Invitrogen, Thermo Fisher). *∆akuB*^KU80^ therefore has two copies of the *pyrG* gene, one mutated, nonfunctional copy at the native *pyrG* locus (d'Enfert et al. 1996^22^) and one functional copy at the *akuB*^KU80^ locus. As expected for strains deficient in NHEJ, the *∆akuB*^KU80^ isolate is moderately sensitive^24^ to the chemical methane methyl sulfonate (MMS).

To render *∆akuB*^KU80^ amenable *pyrG-*mediated gene replacements, a uridine/uracil auxotroph of *∆akuB*^KU80^ was generated by selection on 5-FOA and deposited at the FGSC as A1160.^24^ The mutation in the *pyrG* allele inserted at the *akuB*^KU80^ genomic locus has not been so far characterized by sequencing. A1160 was later further manipulated to obtain a prototrophic *∆akuB*^KU80^ strain named A1160 pyrG+, with which to facilitate the study of antifungal drug transporters via gene replacement strategies using a dominant selection marker.^27^ In order to restore a functional *pyrG* gene at its native locus, A1160 was converted to uridine/uracil prototrophy via targeted insertion of a functional *A. fumigatus pyrG* from CEA10 into the *pyrG* genomic locus.^27^ In later years the isolate was renamed as MFIG001 and utilized as the progenitor isolate for the genome-wide *A. fumigatus* knockout library.^28^ This strain is available via NCPF at Public Health England as part of the transcription factor knockout library.

## ATCC46645 and derivatives

The ATCC46645 Fresenius strain (American Type Culture Collection) was isolated from pus removed via a bronchoscopy from the left bronchus of a patient with an acute febrile respiratory infection^29^ and its first scientific use reported in 1997.^30^

**Figure 4.**
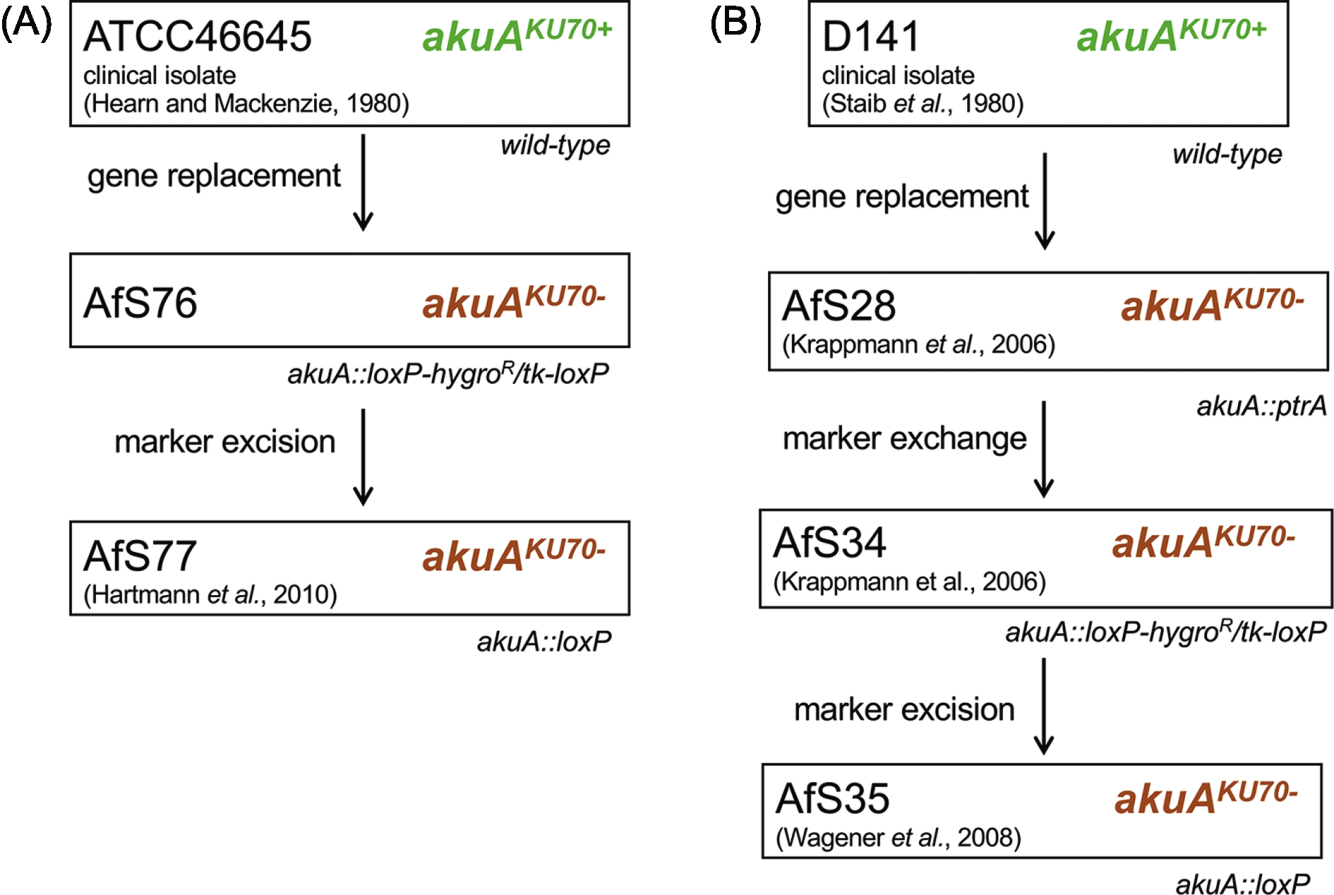
ATCC46645, D141 and derivatives thereof. Diagram depicting strains originating from ATCC46645 **(A)** or D141 **(B)** with respective details on origin and references. The uridine/uracil requirements and the genomic organization of the *akuA*^KU70^ locus are indicated for each isolate within these lineages.

In ATCC46645, the *akuA*^KU70^ gene (identified by identity to *AFUA_5g07740* in Af293 as ATCC46645 does not have lineage specific identifiers), which is essential for the non-homologous end joining machinery, was deleted by replacing its coding sequence with the *loxP-hyg^R^/tk-loxP* marker module to yield the intermediate strain AfS76 (Fig. 4A). AfS76 was in turn modified to construct AfS77/A1280, in which the *loxP-hyg^R^/tk-loxP* cassette was excised via transient Cre expression from pSK215.^31^

Direct comparison of ATCC46645 and CEA10 infectivity in a leukopenic murine model of infection, revealed that a ∼10 times higher inoculum of ATCC46645 was necessary than that required for CEA10 to achieve similar mortality rates.^5^ Nevertheless, low dose infection models (with 5 × 10^3^ conidia) have been established for ATCC46645.^32^

Interestingly, macrophage phagocytosis rates of the ATCC46645 strain are lower than those reported for the CEA10 lineage, perhaps caused by differences in growth rate.^33^

## D141 and derivatives

*A. fumigatus* D141 was isolated by Staib et al. in 1980 from a 45-year old male with an aspergilloma. The patient sample was mainly composed of a white *A. fumigatus* variant mixed with a typical looking greenish *A. fumigatus*, which was named D141^34^ (Fig. 4B). D141 served as a prototrophic, marker/resistance-free wild-type isolate to generate the NHEJ-deficient strain AfS28/A1157^35^ by deleting the *akuA*^KU70^ gene and replacing it with the *ptrA* resistance marker (containing the *ptrA* gene, AO090003000090, from *Aspergillus oryzae* under control of its native promoter, which confers resistance to the antibiotic pyrithiamine).^36^ The genome sequence of AfS28 is available via the JGI genome portal (project ID: 1098580 and 1098483). A derivative of AfS28 is the AfS34/A1158 strain^35^; this strain was selected for hygromycin resistance and pyrithiamine sensitivity after the exchange of the *ptrA* marker with the recyclable *loxP-hyg^R^/tk-loxP* marker cassette.^37^ In AfS34, the *loxP-hyg^R^/tk-loxP* marker was excised by transient expression of the Cre recombinase from the autonomously replicating plasmid pSK215 to result in the marker/resistance-free derivative AfS35/A1159 (*akuA*^KU70^*::loxP*).^38^ This strain was recently sequenced and genomic, metabolomics and infection comparison with the A1163 and CEA10 strains found significant differences in carbon- and nitrogen metabolism, protease secretion, cell wall metabolism and virulence in a triamcinolone murine model of pulmonary aspergillosis.^39^

**Table 1.** Table of isolates.

Name	Genotype	Other denominations	Origin	Source	Sequence accession number	Phenotype	Reference
Af293	Wild type; *MAT1-2*	FGSC A1100, IHEM18963, CBS101355, NCPF7367, MYA-5609	Lung biopsy specimen of neutropenic IPA patient	David Denning Laboratory	GCA_000002655.1	Pyr^+^5-FOA^S^	^3,4^
Af293.1	*pyrG^−^; MAT1-2*	FGSC A1137	Af293	…	…	Pyr^−^5-FOA^R^	^18^
CEA10	Wild type; *MAT1-1*	CBS 144–89CBS 144.89, AF10	Patient with IPA	CBS-KNAW Fungal Biodiversity Centre	…	Pyr^+^5-FOA^S^	^19,20^
CEA17	*pyrG^−^; MAT1-1*	…	CEA10	…	…	Pyr^−^5-FOA^R^	^22^
A1163	*pyrG^−^, pyrG^An^; MAT1-1*	…	CEA17	…	GCA_000150145.1	Pyr^+^5-FOA^S^	^22^
*ΔakuB* ^KU80^	*ΔakuB* ^ku80^::*pyrG^Af^*-*zeo; MAT1-1*	FGSC A1151	CEA17	…	…	Pyr^+^5-FOA^S^MMS^S^Zeo^R^	^24^
A1160^−^	*ΔakuB* ^ku80^::*pyrG^−^*-*zeo; MAT1-1*	…	Δ*akuB*^KU80^	…	…	Pyr^−^5-FOA^R^MMS^S^ (*)Zeo^R^ (*)	^24^
A1160^+^	*ΔakuB* ^ku80^::*pyrG^−^*-*zeo, pyrG^−^::pyrG^Af^; MAT1-1*	A1160p+, MFIG001	A1160^−^	…	…	Pyr^+^5-FOA^S^MMS^S^ (*)Zeo^R^ (*)	^27^
ATCC46645	Wild type*; MAT1-1*	…	Pus from bronchoscopy from the left bronchus of a patient with acute febrile respiratory infection.	…	…	Pyr^+^5-FOA^S^	^29^
AfS77	*ΔakuA*::*loxP; MAT1-1*	FGSC A1280	ATCC46645	…	…	Pyr^+^5-FOA^S^	^31^
D141	Wild type; *MAT1-1*	…	Isolated from an aspergilloma	…	…	Pyr^+^5-FOA^S^	^34^
AfS35	*ΔakuA*::*loxP; MAT1-1*	FGSC A1159	D141	…	…	Pyr^+^5-FOA^S^	^38^
AfS28	*ΔakuA*::*ptrA; MAT1-1*	FGSC A1157	D141	…	…	Pyr^+^5-FOA^S^Ptr^R^	^35^
AfS34	*ΔakuA*::*loxP-hygroR/tk-loxP; MAT1-1*	FGSC A1158	AfS28	…	…	Pyr^+^5-FOA^S^Hygro^R^	^35^

(*) data not available, phenotype inferred from parental isolate of origin. IPA: invasive pulmonary aspergillosis; Pyr^+^ and Pyr^−^ pyrimidine: prototroph or auxotroph, respectively; S and R superscript: ‘sensitive’ and ‘resistant,’ respectively; 5-FOA: 5-fluoro-orotic acid; MMS: methane methyl sulfonate; Ptr: pyrithiamine; Zeo: zeocin; Hygro: Hygromycin.

## Conclusions

An expanding number of studies demonstrate differences between *A. fumigatus* isolate phenotypes,^5,15,39,40^ immune responses^17,33,41^ and virulence in various models of infection.^15,39,42–44^ The use of different experimental conditions and murine models/strains does not always allow exhaustive cross-comparison of the vast amount of virulence data from *A. fumigatus* isolates. However, upon parallel challenge with different clinical isolates, significant interstrain variability was observed with respect to the survival of different model organisms, such as flies,^43^ mice,^15,41,42^ zebrafish,^16^ and waxworm.^44^ Likewise, a wide strain-dependent variation has been documented with regards to macrophage phagocytosis and killing *in vitro*^33,39^ and cytokine production by dendritic cells^41^*in vitro* and/or *in vivo* (using immunocompetent C57BL/6 mice). These findings highlight a crucial need to understand the lineage of strains routinely used in the laboratory and, whenever possible going forward, to construct and test *A. fumigatus* mutants in multiple genetic backgrounds.
